# Angiogenesis gene expression in murine endothelial cells during post-pneumonectomy lung growth

**DOI:** 10.1186/1465-9921-12-98

**Published:** 2011-07-27

**Authors:** Miao Lin, Kenji Chamoto, Barry C Gibney, Grace S Lee, Dinee Collings-Simpson, Jan Houdek, Moritz A Konerding, Akira Tsuda, Steven J Mentzer

**Affiliations:** 1 Laboratory of Adaptive and Regenerative Biology, Brigham & Women's Hospital, Harvard, Medical School, Boston MA, USA; 2 Institute of Functional and Clinical Anatomy, University Medical Center of the Johannes Gutenberg-University Mainz, Germany; 3 Molecular and Integrative Physiological Sciences, Harvard School of Public Health, Boston, MA, USA

## Abstract

Although blood vessel growth occurs readily in the systemic bronchial circulation, angiogenesis in the pulmonary circulation is rare. Compensatory lung growth after pneumonectomy is an experimental model with presumed alveolar capillary angiogenesis. To investigate the genes participating in murine neoalveolarization, we studied the expression of angiogenesis genes in lung endothelial cells. After left pneumonectomy, the remaining right lung was examined on days 3, 6, 14 and 21days after surgery and compared to both no surgery and sham thoracotomy controls. The lungs were enzymatically digested and CD31^+ ^endothelial cells were isolated using flow cytometry cell sorting. The transcriptional profile of the CD31^+ ^endothelial cells was assessed using quantitative real-time polymerase chain reaction (PCR) arrays. Focusing on 84 angiogenesis-associated genes, we identified 22 genes with greater than 4-fold regulation and significantly enhanced transcription (p <.05) within 21 days of pneumonectomy. Cluster analysis of the 22 genes indicated that changes in gene expression did not occur in a single phase, but in at least four waves of gene expression: a wave demonstrating decreased gene expression more than 3 days after pneumonectomy and 3 sequential waves of increased expression on days 6, 14, and 21 after pneumonectomy. These findings indicate that a network of gene interactions contributes to angiogenesis during compensatory lung growth.

## Introduction

In most circumstances, angiogenesis does not occur in the adult pulmonary circulation [1,2]. Although structural adaptations are well-documented in the bronchial circulation [3,4], the evidence for angiogenesis in the pulmonary circulation is sparse [5]. Pulmonary angiogenesis has been demonstrated in a few animal models including biliary cirrhosis [6], chronic Pseudomonas infections [7], metastatic disease [8], and post-pneumonectomy lung growth [9]. The finding that experimental (monocrotaline) pulmonary hypertension induces angiogenesis in the pleura and bronchovascular bundle, but not in the alveolar capillaries [3], underscores the distinctive biology of pulmonary angiogenesis.

Post-pneumonectomy compensatory lung growth is a particularly intriguing example of pulmonary angiogenesis. Within weeks of pneumonectomy, compensatory lung growth has been documented in many mammalian species including rats [10], mice [11] and dogs [12]. Recent evidence indicates that lung growth does not reflect alveolar distension, but an increase in the number of alveoli [13]. Working in the dog model, Hsia and colleagues estimated that the remaining lung after right pneumonectomy increases its capillary blood volume 43% and the capillary surface area 34% [9]. Using a design-based estimate of capillary length [14] and allometric scaling, a comparable increase in mouse lung blood volume implies sufficient angiogenesis for more than 3 km of new pulmonary vessels. The mechanism of this dramatic pulmonary vascular growth remains unclear.

Previous work in murine post-pneumonectomy compensatory lung growth has implicated a diverse set of angiogenic and growth-related genes including epidermal growth factor (*Egr1) *[15], keratinocyte growth factor (*Fgf7) *[16], hepatocyte growth factor (*Hgf *)[17], hypoxia-inducible factor-1α (*Hif1a) *[18], endothelial nitric oxide synthase (*Nos3) *[19], platelet-derived growth factor β *(Pdgfb *[20], and vascular endothelial growth factor (*Vegfa) *[21]. Attempts to define transcriptional regulation using microarrays and bulk RNA, however, have identified few genes clearly associated with capillary angiogenesis [22,23].

The complex morphogenetic changes in the growing lung include epithelial and stromal growth as well as pulmonary vascular angiogenesis. This dynamic process of tissue morphogenesis suggests a coordinated process involving complex network interactions and intercellular signaling. In this report, we study a transcriptional signaling of a central component pulmonary angiogenesis; namely, the pulmonary endothelium.

## Methods

### Mice

Male C57/B6 mice (Jackson Laboratory, Bar Harbor, Maine), 25 to 33 gm, were used in all experiments. The care of the animals was consistent with guidelines of the American Association for Accreditation of Laboratory Animal Care (Bethesda, MD); all animal protocols were reviewed and approved by the Institutional Care and Use Committee.

### Gene expression study design

The age-matched mice received identical care prior to selection for one of three experimental groups: 1) no surgery control, 2) sham thoracotomy control, and 3) left pneumonectomy. Experimental time points were at Day 0, 3, 6, 14 and 21 days. Day 0 studied gene expression in the right lung without any prior surgery (sham thoracotomy or left pneumonectomy)(N = 15 mice). Days 3, 6, 14 and 21 compared the right lung after left pneumonectomy with no surgery controls (N ≥ 9 mice each time point). Day 14, the day reflecting expression of the peak number of statistically significant genes, compared all three experimental groups (left pneumonectomy, sham thoracotomy and no surgery controls)(N = 9 mice per condition). In each experimental group, enzymatic digestion of the right lung was followed by isolation of the CD31^+^CD11b^- ^"endothelial cells" by flow cytometry cell sorting. In some experiments, three parameter (CD31^+^CD11b^-^CD45^-^) cell sorting was used. For the endothelial analysis presented, no significant difference between 2 and 3 parameter sorting was identified. RNA was isolated from the endothelial cells and used for characterizing angiogenesis-related gene expression.

### Pneumonectomy

After anesthesia and intubation, the animal was ventilated on a Flexivent (SciReq, Montreal, QC Canada) at ventilator settings of 200/min, 10 ml/kg, and PEEP of 2 cmH_2_O with a pressure limited constant flow profile [24]. A thoracotomy was created in the left fifth intercostal space. The hilum was ligated with a 5-0 surgical silk tie (Ethicon, Somerville, NJ) and the lung sharply excised. A recruitment maneuver involving a 3 sec ramp to 30 cmH_2_O and a 3 sec plateau was performed while closing the thoracotomy with a 3-0 silk stitch (Ethicon). At the completion of the procedure, the animal was removed from the ventilator, maintained on a warming blanket and observed for spontaneous ventilation. The FlexiVent (SCIREQ) system was used to determine lung volumes at a 30 cmH_2_O inflation pressure [24].

### Immunohistochemical staining

Cryostat sections were obtained from lung specimens perfused with O.C.T. compound and snap frozen. The slides were serum blocked and treated with anti-Ki-67 (Clone TEC-3, Dako, Hamburg, Germany) monoclonal antibodies (mAb) at 10-20 ug/m for one hour. After rinsing, the anti-Ki-67 binding was detected with an avidin-biotin-peroxidase complex (Vectastatin ABC-Kit, Vector Laboratories) or with the Envision^® ^kit (Dako) and counter-stained with hematoxylin.

### Lung digestion

The lung was processed in a modification of a procedure previously described [25]. Briefly, the lung was harvested at the airway to minimize extra pulmonary airway. The lung parenchyma was minced into 1 mm^3 ^pieces and processed by enzymatic digestion: 1 mg/ml collagenase (Sigma, St. Louis, MO) and 2.5 U/ml dispase solution (Collaborative Biomedical Products, Bedford MA). The suspension was incubated at 37°C on a rotary shaker for 40 min. The lung was triturated using an 18 g needle and filtered through a 70 um nylon mesh screen (BD Falcon, Bedford, MA) prior washing in serum containing RPMI-1640 medium (Thermo scientific, Pittsburgh, PA). Cells were treated with red blood cell lysis buffer (BD Pharm Lyse, BD Biosciences) diluted 1:10 in H_2_O and used at the concentration of 1-3 × 10^7^/ml.

### Cell count and calculation

The digested lung cells were counted using a Neubauer hemacytometer (Fisher, Pittsburgh, PA). Dead cells were excluded by trypan blue (Sigma, St Louis, MO). The number of CD31^+ ^cells was calculated by using flow cytometric analysis: (CD31^+ ^cell number) = (total lung cell number) × (% of CD31^+ ^cells among total cells)/100.

### DNA cell-cycle analysis

For cell cycle analysis, the digested lung cells were stained at 1-2 × 106 cells/ml with 10 uM Hoechst 33342 (Invitrogen, Carlsbad, CA). After defining ModFit parameters (Modfit, Verity Software House, Topsham ME), the viable lung cells were gated based on forward and side scatter parameters to exclude debris. In each experiment, the digested lung cells were stained at 1-2 × 10^6 ^cells/ml with 10 uM Hoechst 33342 (Invitrogen, Carlsbad, CA) in media containing 2% fetal calf serum at pH7.2 for 60 minutes. The cells were immediately analyzed using a tri- excitation laser (407 nm, 488 nm and 633 nm ex) and a FACSCanto II flow cytometer (BD Biosciences, Franklin Lakes, NJ). The samples were individually assessed to be within the guidelines of the DNA Consensus Conference criteria for quality (extrapolated to non-neoplastic tissue)[26]. After defining ModFit parameters, the viable lung cells were gated based on forward and side scatter parameters to exclude debris.The cells were analyzed by the ModFit autoanalysis and autolinearity algorithms (Verity Software House, Topsham ME). Because of nuclear density interference and other staining nonlinearities [27], the G2/G1 ratio was typically modified using the ModFit autolinearity algorithm. Autolinearity G2/G1 ratios ranged from 1.93-1.99. ModFit estimates of aggregates was 4.9 ± 4.0% and debris was 13.4 ± 5.8% of total events. The mean number of all cycle events was 74,996 ± 12750 and the mean number of modeled events was 90,471 ± 10359.

### Flow cytometry

For phenotypic analysis, the digested lung cells were incubated with a 5-fold excess of directly conjugated fluorescein isothiocynate (FITC) or phycoerythrin (PE) anti-mouse antibodies with isotype controls: anti-CD31 (rat IgG2a, Clone 390, eBioscience)[28], anti-CD11b (rat IgG2b, Clone M1/70, BD Bioscience)[29] and anti-CD45 (rat IgG2b, Clone 30-F11, eBioscience)[30] prior to analysis using a tri-laser flow cytometer (BD FACSAria and FACSCanto II (BD Biosciences) with tri excitation laser (407 nm, 488 nm and 633 nm ex). The data were analyzed by FCS Express 4 software (De Novo Software, Los Angeles, CA). In all analyses, debris were eliminated by gating the alive cell population of side and forward light scatter and further by gating 7AAD (BD Biosciences)-negative population as previously described [31]. In most experiments, the analysis was based on 100,000 events.

### RNA and quantitative PCR

In all RNA isolations, the total RNA quality was assessed by using an Agilent 2100 Bioanalyzer (Agilent Technologies, Palo Alto, CA). RNA integrity numbers (RIN) of the RNA samples were uniformly greater than 7.3 (mean 8.5; range 7.3 to 9.8)[32]. Real-time PCR was performed with SYBR green qPCR master mixes that include a chemically-modified hot start Taq DNA polymerase (SABiosciences, Frederick, MD). PCR was performed using an ABI Prism 7300 Real-Time PCR System (Applied Biosystems).

### PCR arrays

The commercially available PCR arrays, obtained from S.A. Biosciences (Frederick, MD), included the Endothelial Cell Biology Array (PAMM-015), Inflammatory Cytokines and Receptors Array (PAMM-011), Dendritic & Antigen Presenting Cell Array (PAMM-406) and the Angiogenesis Array (catalog PAMM-024). Real-time PCR was performed with SYBR green qPCR master mixes that include a chemically-modified hot start Taq DNA polymerase (SABioscience). PCR was performed on ABI 7300 Real-Time PCR System (Applied Biosystems, Carlsbad, CA). For all reactions, the thermal cycling conditions were 95°C for 10 min followed by 40 cycles of denaturation at 95°C for 15 sec and simultaneous annealing and extension at 60°C for 1 min. The two sets of triplicate control wells (RTC and PPC) were also examined for inter-well and intra-plate consistency; standard deviations of the triplicate wells were uniformly less than 1C_t_. The variance of genes common to the 4 arrays were uniformly less than 0.5C_t_. To reduce variance and improve inferences per array [33], a design strategy was used that combined pooled samples (3-4 mice per array).

### Statistics and bioinformatics

Our quantitative PCR assumed that DNA template and/or sampling errors were the same for all amplifications; our internal control replicates indicated that our sample size was sufficiently large that sampling errors were statistically negligible [34]. The exponential phase of the reaction was determined by a statistical threshold (10 standard deviations). Flow cytometry statistical analysis was based on measurements in at least three different mice. The unpaired Student's t-test for samples of unequal variances was used to calculate statistical significance. The data was expressed as mean ± one standard deviation. The significance level for the sample distribution was defined as P <.05. Clustering of the statistically significant genes (t-test; p <.05) was performed using an agglomerative hierarchical clustering algorithm [35,36].

## Results

### Post-pneumonectomy lung growth

**Figure 1:**
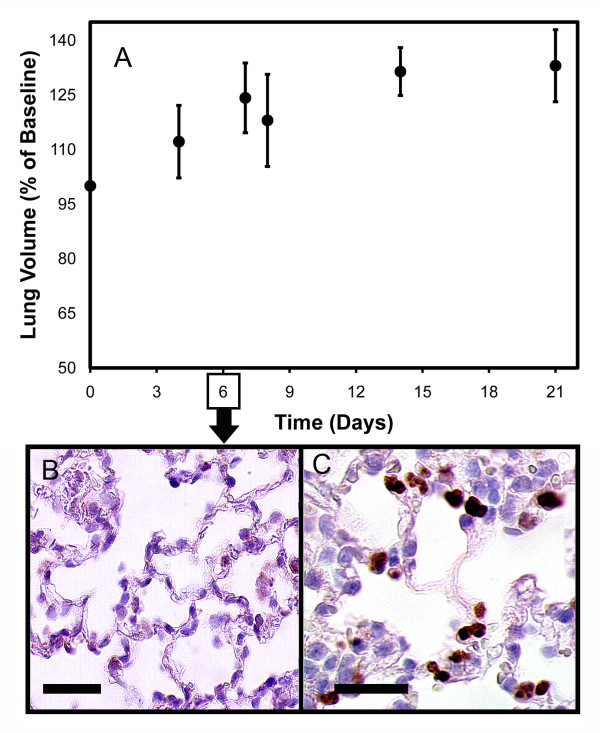
**Functional and histologic evidence of compensatory lung growth after pneumonectomy (Day 0)**. A) Lung volumes obtained by a FlexiVent maximal vital capacity maneuver (named TLC by SCIREQ). The mouse was allowed to exhale to residual volume then ventilated with a 3 sec ramp to a 3 sec plateau at 30 cmH_2_O pressure. The measured volume was expressed as a percent of Day 0 post-pneumonectomy baseline. Each data point reflects the mean of N = 3 mice ± 1 SD; 3 TLC maneuvers per mouse. B) Control and C) Ki-67 (Clone TEC-3, Dako, Hamburg, Germany) immunohistochemistry of the lung during the "growth" phase (Day 6) demonstrated scattered positive cells providing evidence of proliferation within the alveolar septa (arrow, bar = 25 um).

To confirm a compensatory increase in lung volume after pulmonary resection, we studied the pulmonary mechanics of mice on Days 3, 6, 14, and 21 after left pneumonectomy. Using the FlexiVent rodent ventilator, the maximal vital capacity recruitment maneuver (referred to as the "TLC" volume by SciReq) demonstrated a statistically significant increase in lung volumes within 2 weeks of surgery (Figure 1A; p <.01). During the phase of greatest change in volume, immunohistochemistry of the lung using antibodies against the Ki-67 cell cycle protein demonstrated staining in the alveolar septa (Figure 1C). Nuclei positive for the Ki-67 protein were evident in both the alveolar septa and juxta-alveolar interstitium likely reflecting cell cycle activity in many lung cell types.

### Endothelial cells after pneumonectomy

**Figure 2:**
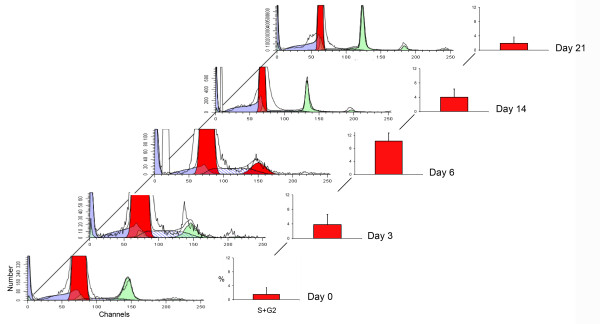
**Cell cycle profiling of post-pneumonectomy lung CD31^+ ^endothelial cells obtained by enzymatic digestion and analyzed using flow cytometry (ModFit, Verity Software House)**. Automated analysis of the ModFit model components, processed by a Marquardt nonlinear least-squares analysis, excluded aggregates (green) and identified S phase (blue hatched area) and G2 phase (second red peak) cells at five time points (Day 0, 3, 6, 14 and 21). The combined analysis of the percentage of cells in S+G2 phase of the cell cycle at various time points after pneumonectomy in the column chart (right; mean ± SD; 4-6 mice per time point).

**Figure 3:**
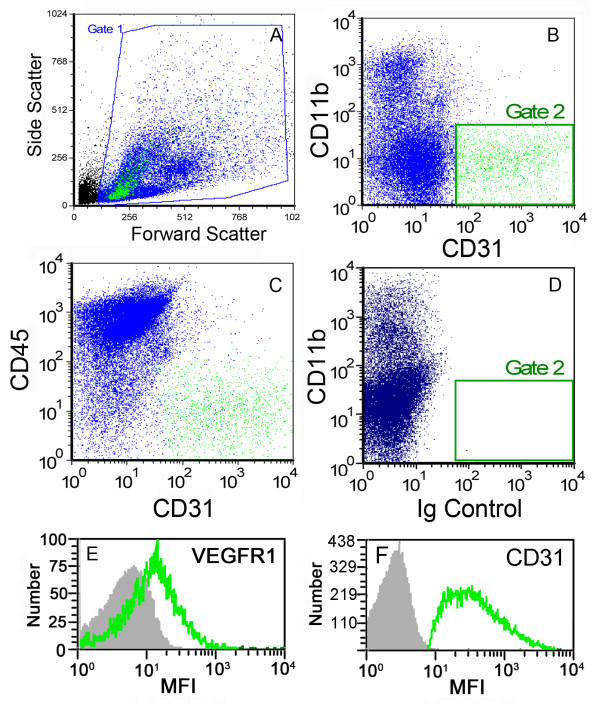
**Phenotype of lung endothelial cells after enzymatic digestion**. A) Flow cytometry light scatter characteristics of the digested lung cells. Gate 1 was used for subsequent dual parameter analyses. The green population in panels A-D reflect Gate 2 defined in panel B. B) Dual parameter histogram of lung cells stained with directly conjugated anti-CD31 (390, FITC) and anti-CD11b (M1/70, PE) monoclonal antibodies. C) Consistent with an endothelial phenotype, the CD31^+^/CD11b^- ^cells in Gate 2 were also negative for the leukocyte antigen CD45 (30-F11, PE). D) Isotype control of the anti-CD31 antibody. E-F) Surface expression of the *Flt1 *(VEGFR1) and *Pecam1 *(CD31) gene products of the endothelial cell population presented as a single parameter histogram. Gray reflects the surface staining of the isotype control of each antibody; MFI = mean fluorescence intensity. The CD31^+ ^cells (Gate 2) were defined as endothelial cells for subsequent experiments.

A quantitative assessment of the time course of cell proliferation was obtained using cell cycle flow cytometry of CD31^+ ^endothelial cells (Figure 2). An early increase in S-phase cells was noted on Day 3 after pneumonectomy. By Day 6 after pneumonectomy, 10% of the cells in the right lung were in either the S or G2 phases of the cell cycle. To investigate the endothelial response to pneumonectomy, the lung digests were analyzed by flow cytometry. Baseline analysis by flow cytometry demonstrated 8-15% of the cells were positive for the endothelial cell surface molecule CD31 (PECAM-1) and negative for leukocyte markers CD11b and CD45 (Figure 3A-D). As expected, the endothelial cells uniformly expressed the surface molecules VEGFR1 (*Flt1*) and CD31(*Pecam1*) (Figure 3E-F).

**Figure 4:**
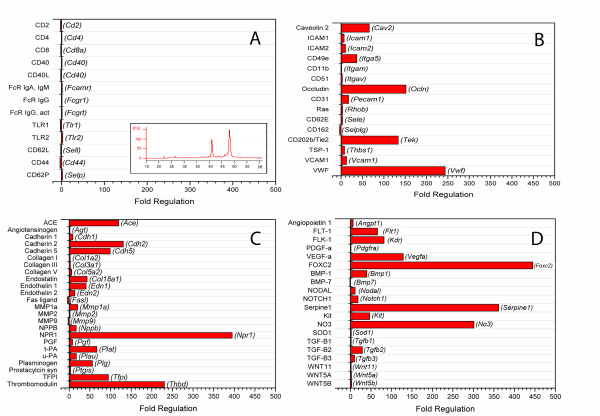
**Representative gene transcription profile of CD31**^**+ **^**cells**. Baseline (control) lungs were enzymatically digested and the CD31^+ ^cells isolated by flow cytometry cell sorting. The RNA extracted from CD31^+ ^cells consistently demonstrated RIN greater than 8 (inset: representative RNA electropherogram). The CD31^+ ^cells were compared to CD31^-^/CD11b^- ^cells on multiple PCR arrays (PAMM-015, PAMM-011 and PAMM-406, SABiosciences). The CD31^+ ^cell gene transcription profile, expressed as the fold regulation (Log_2_), was grouped by phenotypic and functional associations: A) leukocyte membrane molecules, B) endothelial cell function-associated molecules, C) extracellular matrix molecules and D) angiogenesis and growth factor molecules. Data representative of N = 16 mice.

To confirm the cells isolated by flow cytometry were endothelial cells, the transcriptional profile was assessed by PCR arrays. Of note, the isolated RNA was consistently negative for leukocyte genes (Figure 4A), but positive for genes associated with endothelial cell function-associated molecules, extracellular matrix molecules and angiogenesis and growth factor molecules (Figure 4B-D).

### Angiogenesis-related transcription

**Figure 5:**
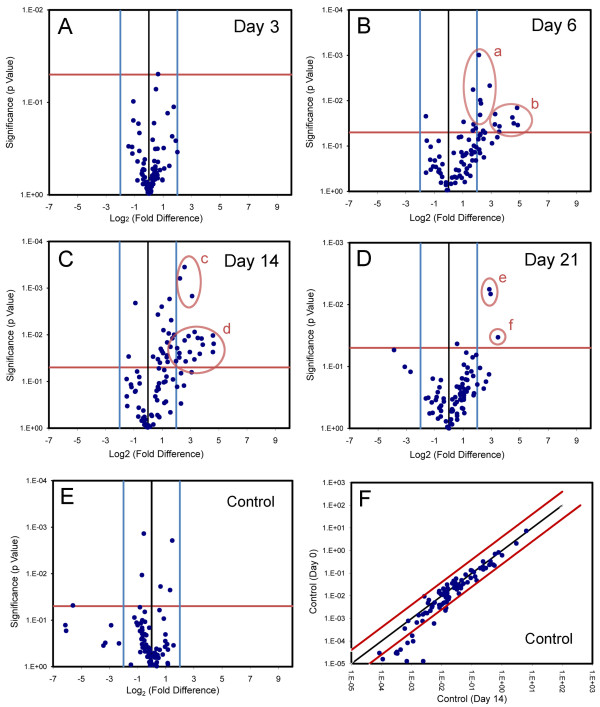
**Expression of angiogenesis-related genes in CD31**^**+ **^**cells after pneumonectomy**. A-D) Gene expression in mice 3, 6, 14 and 21 days after pneumonectomy was compared to age-matched controls without surgery. The log_2 _fold-change in gene expression was plotted against the p-value (t-test) to produce a "volcano plot." The vertical threshold reflected the relative statistical significance (red horizontal line, -log_10_, p < 0.05); the horizontal threshold reflected the relative fold-change in gene expression (blue vertical line, 4-fold). The significantly up-regulated expression of specific genes (red elipses): a) *Tnfaip2, Plg, Anpep, Pgf, Vegfb *and *Thbs2*; b) *Csf3, Col4a3, Sphk1, Ereg, Vegfa, Lect1, Col18a1 *and *Il6*; c) *Ereg, Ephb4 *and *Vegfa*; d) *Tbx1, Cxcl5, Col4a3, Col18a1, F2, Vegfc, Sphk1, Ccl11, S1pr1, F2, Il6, Lect1, Flt1 *and *Csf3*. e) *Sphk1 *and *Vegfa*; f) *Csf3*. Each data point reflects triplicate or quadruplicate arrays of 9 to 21 mice (Day 0, N = 15 mice; Day 3, N = 9 mice; Day 6 = 9 mice; Day 14, N = 9 mice; Day 21, N = 21 mice). E) Indicating a limited impact of the thoracotomy alone, the volcano plot comparison of sham thoracotomy and no surgery control demonstrated no significant change in gene expression on Day 14 after pneumonectomy. F) Scattergram indicating the stability of the control cell population (CD11b^- ^CD31^- ^) used for gene expression analysis. Angiogenesis gene expression in the CD11b^-^CD31^- ^cells was compared on Day 0 and Day 14 after pneumonectomy (N = 3 mice, each time point). Gene expression demonstrated only one gene (*Bai1*) with significantly decreased expression on Day 14 after pneumonectomy.

To investigate the endothelial response to pneumonectomy, the transcriptional profile of the CD31^+ ^cells on Days 3, 6, 14 and 21 after pneumonectomy was studied by PCR arrays. Volcano plots were used to identify gene transcription significantly increased or decreased relative to age-matched or sham thoracotomy control mice. At 3 days after pneumonectomy, no genes were significantly different from control (p <.05) with expression levels >4-fold controls; however, analyses 6 days after pneumonectomy identified 14 genes with differentially increased transcription (Figure 5B). Similarly, 17 genes demonstrated enhanced transcription 14 days after pneumonectomy (Figure 5C). In contrast, the RNA obtained from CD31^+ ^cells 21 days after pneumonectomy demonstrated only 3 genes with increased expression (Figure 5D). Expression of 9 genes was increased at two or more time points (*Col18a1, Col4a3, Csf3, Ereg, F2, Il6, Lect1, Sphk1, *and *Vegfa*). None of the genes with decreased expression after pneumonectomy reached statistical significance with a >4-fold change. Of note, there was no significant difference in gene expression when sham thoracotomy and the no surgery controls were compared 14 days after pneumonectomy (Figure 5E). Similarly, the CD11b^- ^CD31^- ^cells used for comparison in the gene expression analysis demonstrated temporal stability; there was little change in angiogenesis gene expression when the Day 0 and Day 14 post-pneumonectomy arrays were compared (Figure 5F).

### Temporal expression pattern

**Figure 6:**
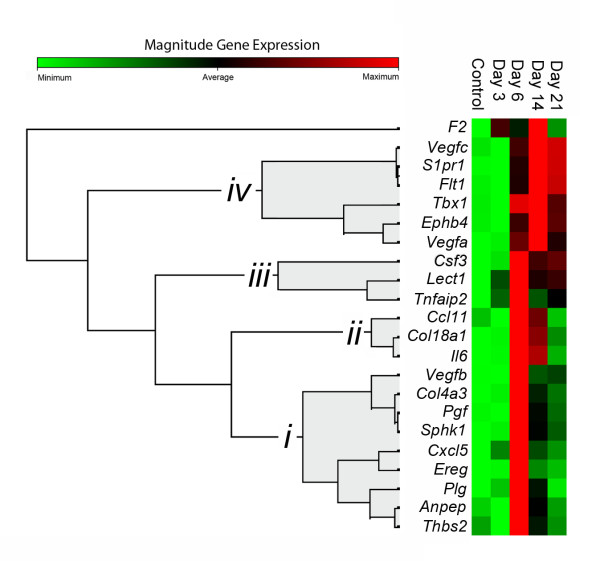
**Temporal expression clustergram mapping of angiogenesis genes**. Genes demonstrating a statistically significant increase in expression (p <.05, t-test) at one or more time points were clustered using an agglomerative hierarchical clustering algorithm. The similarity/dissimilarity metric, based on the Pearson correlation coefficient between two dimension profiles of qRT-PCR gene expression, identified 4 waves (*i-iv*) of gene expression. The dendrogram was based on average linkage clustering. The colored matrix display was encoded based on the value of the gene at the 5 time points; the relative dissimilarity index in the unshaded portion of the dendrogram was compressed for presentation purposes.

The temporal patterning of the 84 genes in the angiogenesis PCR array was investigated using hierarchical clustering. Because of the wide variation in quantitative gene expression, we used pairwise Pearson correlation coefficients to assess similarity/dissimilarity. The genes selected for clustering were 22 genes that demonstrated both a >4-fold change and a statistically significant (p <.05) difference from control mice (Figure 6). Using this approach, gene expression clusters demonstrated unexpected similarities in genes such as *Anpep *and *Thbs2*, as well as functionally more predictable similarities in genes such as *Ephb4 *and *Vegfa*. Consistent with phases or "waves" of gene expression [37], distinct patterns were identified on Days 3, 6, 14 and 21. Most genes demonstrated a single wave of expression; only *Csf3 *demonstrated a bimodal pattern with expression peaks on Days 6 and 21.

## Discussion

In this report, we studied the expression of angiogenesis genes in isolated endothelial cells during murine post-pneumonectomy lung growth. The quantitative profiling of 84 angiogenesis-associated genes suggested several conclusions. First, pneumonectomy resulted in a sustained transcriptional response in the lung endothelial cells. The persistent change in the expression of multiple angiogenesis-associated genes suggested a transcriptional "state" that persisted substantially longer than the 7-14 day growth period. Second, the statistically significant change in the expression of 22 angiogenesis-associated genes indicated that alveolar capillary angiogenesis did not depend upon a single dominant or controlling genetic element, but a complex sequence of gene expression. An understanding of these temporal dynamics will likely be necessary for effective therapeutic control. Finally, the use of an isolated cell population provided transcriptional evidence of intercellular signaling. The expression of genes known to participate in receptor-ligand pairs suggested testable predictions in complementary cell types.

A contribution of this study is the "tissue-scale" approach to post-pneumonectomy alveolar construction. Tissue-scale models typically consider individual cell types as "nodes" and the exchanges between cells as "edges" in computational networks. The structural composition of the lung--repeating functional units and relatively uniform cell types--makes this approach particularly appealing in the study of compensatory growth. In tissue-scale networks, any measurable or quantifiable property can be considered a variable of interest to be associated with a node. In adult morphogenesis, issues such as cell shape properties and the mechanical milieu are also among the variables associated with a node at this scale. Here, we studied a phenotypically uniform cell type (endothelial cells) and analyzed the transcriptional profile associated with the morphogenetic process of angiogenesis. We anticipate that the value of this data will grow as 1) other nodes in the tissue-scale network are defined, and 2) more endothelial cell variables are characterized.

Our data indicate that compensatory growth was not associated with a single transcriptional wave, but a temporal pattern of gene expression. The concept of a temporal dynamics of gene expression has been explored in many developmental studies [38,39]. In most cases, the patterns of gene expression are presumed to reflect a largely context-insensitive linear cascade of genes with sequentially ordered expression [40]. An alternative interpretation is that the temporal dynamics reflect network interactions with intercellular signaling and feedback control. For example, the enhanced expression of *Ereg *(epigregulin) in endothelial cells suggests a complementary target receptor on epithelial cells. *Ereg *is a member of the epidermal growth factor family and can function as a ligand of EGFR (epidermal growth factor receptor), as well as a ligand of most members of the ERBB (v-erb-b2 oncogene homolog) family of tyrosine-kinase receptors. Since EGFR is expressed on all epithelial and stromal cells [41], the transcription of *Ereg *by endothelial cells suggests a growth promoting interaction between these cell types. Perhaps relevant to the post-pneumonectomy milieu, *Ereg *has been implicated in smooth muscle proliferation and differentiation [41], mechanical strain [42] and wound healing [43]. We predict that future studies of isolated epithelial cells will demonstrate the coexpression of complementary receptor-ligand gene pairs.

The number of identifiable waves of gene expression was a consequence of our experimental design: we studied lungs 3, 6, 14 and 21 days after pneumonectomy. The time points were chosen to reflect the tempo of angiogenesis in the developing rat lung. Burri et al. have described several phases of postnatal lung growth within 21 days: expansion of the lung, rapid alveolarization, and septal restructuring [44,45]. To provide an overview of the entire growth period, we have defined the transcriptional pattern over this 21 day period. An alternative time course is suggested by morphometric studies [13]. In these investigations, the authors demonstrated a significant increase in alveolar number by 6 days after pneumonectomy. A goal of future studies will be to concentrate on the earlier phase of endothelial cell transcription (0-6 days) and enhance the resolution of the pattern described here.

Our findings have several practical limitations. Foremost, we used quantitative PCR arrays to identify statistical significance (t-test) within and between time points, but de-emphasized the fold regulation of gene expression. Contemporary qRT-PCR arrays provide a quantitative assessment of gene expression relative to housekeeping genes (ΔC_t_) and control or calibrator samples (ΔΔC_t_) [46]. Although this method obviates the need for producing a standard curve for each gene, "fold regulation" can be misleading. For example, the kinase insert domain receptor *Kdr *encodes the VEGFR2 receptor and functions as an important mediator of VEGF-induced endothelial proliferation [47]. Post-pneumonectomy *Kdr *expression was only 2-3-fold higher than non-pneumonectomy controls, but 80-fold higher in endothelial cells than in the CD31^-^CD11b^- ^(primarily epithelial cells) controls. Thus, the biological implications of a 2-fold change in a highly transcribed gene are likely to be substantially different from a 2-fold change in a gene with little or no baseline transcription. Because of the limitations of "fold regulation," we emphasized the patterns of expression rather than absolute levels of expression. We used temporal co-expression as a tool for identifying functional relationships. Although our temporal clustering analysis was based on simple correlation coefficients, patterns of expression over time can lead to more sophisticated approaches, such as time-lagged correlations, to infer functional relationships [48].

Flow cytometry, an instrument that uniquely analyzes samples on a per cell basis, provided several advantages in our study. First, flow cytometry permitted the isolation of a large population of phenotypically uniform endothelial cells; that is, cells central to both the functional regulation and structural development of new blood vessels. Second, simultaneous analysis and sorting by flow cytometry provided a link between molecular expression and gene transcription [36]. In this report, we limited our analysis to *Pecam1 *and *Flt1 *gene products--namely, the CD31 and VEGFR1 membrane molecules--but a similar approach is applicable to many of the genes identified in the PCR arrays. Third, since the cell membrane defines a basic regulatory unit of the genome, sorting endothelial cells approximated a uniform transcriptional network [49]. Sorting a population of endothelial cells, rather than using bulk tissue, is analogous to studying the relatively uniform cell populations that have facilitated insights into transcriptional programs in bacteria [50].

The dominant wave of gene transcription occurred on Day 6 after pneumonectomy. Angiogenesis genes such as *Ereg*, *Lect1*, *Plg*, and *Csf3 *demonstrated a significant increase in expression on Day 6 (6- to 30-fold). Interestingly, *Vegfa *expression continued to rise slightly until Day 14--resulting in a slightly misleading temporal clustergram. *Vegfa *has been previously associated with pulmonary capillary development; the selective inactivation of the *Vegfa *gene results in almost complete absence of pulmonary capillaries [51]. The increased expression of *Vegfa *in endothelial cells, however, suggested the possibility of an autocrine effect of *Vegfa *[52] or a trophic influence on other components of the regenerating alveolus. VEGF effects on alveolar type II cells include the activation of cell proliferation, the stimulation of surfactant production [53] and the inhibition of apoptosis [54]. VEGFR1, a membrane receptor stimulated by both VEGFA and VEGFB, has been implicated in the positive regulation of monocyte and macrophage migration [55].

Finally, expression of genes associated with the regulation of angiogenesis also occurred on Days 6 and 14 after pneumonectomy. Angiogenesis genes such as *Col18a*, *Col4a3 and Plg *are known to participate in angiogenesis, but are also the precursors to angiogenesis inhibitors [56]. For example, endostatin is the proteolytic cleavage protein of collagen XVIII and angiostatin is derived from plasminogen [57]. Similarly, the C-terminal cleavage products of collagen IV α1, α2 and α3 possess anti-angiogenic activities; the most notable example is the fragment of the α3 chain of type IV collagen referred to as tumstatin [58]. The varied expression of these genes indicates that post-pneumonectomy angiogenesis involves an interactive and interdependent network of both positive and negative signals; a process likely involving feedback control.

In summary, we have used a "tissue-scale" approach to investigate endothelial cell participation in post-pneumonectomy lung growth. By using flow cytometry to isolate CD31^+ ^endothelial cells and PCR arrays to quantify gene expression, we have 1) contributed gene expression data to the endothelial cell "node" in tissue-scale growth networks, and 2) provided insights into the process of alveolar angiogenesis. Analysis of gene expression in the endothelial cell "node" did not demonstrate a single regenerative signal but a temporal pattern of gene expression. The known physiologic functions of the expressed genes suggest that gene expression does not reflect a pre-programmed response, but a highly interactive and interdependent signaling network. To test our predictions, future work will define other cell types ("nodes") and the exchanges between cells ("edges") for subsequent analysis in computational networks of post-pneumonectomy lung growth.

## Competing interests

The authors declare that they have no competing interests.

## Authors' contributions

Author contribution: All authors have read and approved the manuscript. ML and KC supervised the flow cytometry and PCR experiments; BCG and JH performed the pneumonectomies; GSL and DCS performed the experiments and analyzed the data. MAK, AT and SJM contributed to experimental design, data analysis and manuscript development.

## Abbreviations

Ct: cycle threshold; EGFR: epidermal growth factor receptor; FITC: fluorescein isothiocynate; mAb: monoclonal antibodies; PBS: phosphate buffered saline; PCR: polymerase chain reaction; PEEP: positive end expiratory pressure; PE: phycoerythrin; PPC: positive PCR controls; qRT-PCR: quantitative real-time PCR; RIN: RNA integrity number; RTC: reverse transcription controls; SD: standard deviation
